# Constructing Constraint-Based Simulation System for Creating Emergency Evacuation Plans: A Case of an Outpatient Chemotherapy Area at a Cancer Medical Center

**DOI:** 10.3390/healthcare8020137

**Published:** 2020-05-20

**Authors:** I-Chen Wu, Yi-Chun Lin, Huey-Wen Yien, Fuh-Yuan Shih

**Affiliations:** 1 Department of Civil Engineering, National Kaohsiung University of Science and Technology, Kaohsiung 80778, Taiwan; x791220@gmail.com; 2 YongLin Healthcare Foundation, New Taipei 23143, Taiwan; hwyiin@gmail.com; 3 Department of Emergency Medicine, National Taiwan University Hospital, Taipei 10002, Taiwan; fystone@ntuh.gov.tw

**Keywords:** evacuation plan, outpatient chemotherapy area, constraint-based simulation, building information modeling, agent-based modeling

## Abstract

Making emergency evacuation plans for disaster prevention is always a high priority for hospital administrators to ensure the safety of patients and employees. This study employs the outpatient chemotherapy area of a cancer medical center as an example, and its area involves professional medical care and relatively complex human group behaviors. Hence, it is necessary to simulate evacuations in advance to formulate a special evacuation plan. To achieve this task, a constraint-based simulation system is developed with three major processes: defining spatial and activity constraints, agent-based modeling, and optimizing resource allocation. The spatial boundaries are converted from a three-dimensional model in the Building Information Modeling (BIM) to conduct a visualized simulation. Based on the spatial boundaries, the activities of the agents are set to obey the process specified by work studies. Finally, the Monte Carlo method is employed to simulate the stochastic rescue behaviors of nurses during disasters to determine the fittest resource allocation with the shortest evacuation time for different numbers of patients. The results reveal that the proposed system can output a suggested list of resource allocations and visualized results for administrators when making evacuation plans such that all the people in the area can be safely evacuated.

## 1. Introduction

A hospital is a highly sensitive environment because the behavioral complexity and disaster seriousness of hospitals are not characteristic of those in a general building. In addition to providing high-quality medical services, hospital administrations have to ensure the safety of all patients, employees, and visitors. Notably, sudden disasters always threaten patients’ lives because some patients are unable to move without assistance. It is thus important for hospital administrators to make and drill emergency evacuation plans for disaster prevention. Nevertheless, this task is considered time-consuming and labor-intensive. Moreover, whenever internal layouts are altered to utilize space differently, administrators must check the feasibility of current plans or create new ones. Therefore, obtaining the possible results of evacuation plans in advance would be helpful to administrators when they are determining the final plans. In particular, a chemotherapy area involves several professional medical fields and complex human group behaviors, which together make the evacuation plans for a chemotherapy area more difficult than those of other areas to evaluate and practice. For example, evacuation planners must consider controlling restrictions related to toxicity and the movement limitations of patients undergoing treatment in the chemotherapy area. Accordingly, hospital administrators must develop more specific evacuation plans for outpatient chemotherapy areas as compared to those for other areas of the hospital.

## 2. Related Work

According to Hicks and Glick [1], previous studies related to hospital emergency evacuation can be classified into six major categories: (1) General case reports; (2) Specialized case reports; (3) Review articles that summarize aspects of hospital evacuations, such as why hospitals evacuate; (4) Simulated drills; (5) Simulation models; (6) Broad guidelines. For example, Wabo et al. [2] determined the predictable risks of hospital evacuation, explored the gaps in different evacuation plans, and made similar plans as the guidelines for the evacuation plans of hospitals. Golmohammadi and Shimshak [3] estimated evacuation times for various resource allocations in different hospitals by using the required parameters collected from nineteen hospital evacuation situations. In addition, some studies have evaluated the feasibility of hospital evacuation plans. For instance, Loria et al. [4] examined the actual testing of the evacuation plan and the professional training for emergency evacuation for a hospital in India that had a fire disaster that caused the deaths of most of the critically ill patients in 2011. Jiang et al. [5] indicated that inappropriate evacuation actions in a hospital may cause crisis-related deaths and suggested that an evacuation plan should be developed according to the individual behaviors of patients. Currently, most hospitals in developing countries still train their employees in outdoor spaces and obtain feedback through scenario simulations. Such large-scale practices are not only time consuming and labor intensive but also costly. However, in most cases, hospital administrators develop their evacuation plans based on floor plans, broad guidelines, previous evacuation experience, and case reports [6,7]. Another possible option is to conduct simulations of all possible evacuation plans to obtain their corresponding evacuation times [8]. Nevertheless, the above-mentioned studies did not consider some or any of the following items: (1) The physical reactions caused by the psychological factors of the relevant personnel when the disaster occurs; (2) the impact of a large crowd and space restrictions on evacuation; (3) the extent of additional manpower and resources invested to assist in disaster relief; (4) the fact that stopping an operation to perform an emergency evacuation drill is difficult, so fully understanding and evaluating the actual situation of the evacuation of an actual hospital is also impossible; (5) the need to consider urgent measures, especially in spaces such as chemotherapy areas, to reduce patients’ vulnerability and to prevent the uncontrolled release of toxic substances.

Many people have the feeling that human behavior is "chaotic" or at least very irregular and not predictable. Therefore, social force models [9] are extensively applied to examine evacuation plans. For instance, Han and Liu [10] introduced a social force model into a crowd evacuation simulation to effectively shorten the evacuation time and efficiently improve searches for the best evacuation plans. Sticco et al. [11] employed social force models to simulate safe evacuations under exit restrictions in a case of a panic situation, and their results indicated that a 2.4-m-wide exit can reduce evacuation time by half and that two very close exits can cause an evacuation blockage. Li et al. [12] adopted the social force model and grid density with weight relations to simulate human group behaviors, and their results showed that crowd density, rather than distance, is the main factor that affects the evacuation process when people are in a competitive or panic situation. The agent-based modeling (ABM) [13,14] technique is also widely adopted to simulate real social conditions and human psychological reactions to determine the problems that may arise. ABM provides a microscopic model that uses a social structure to continuously process the time and space of a given event. For example, Joo et al. [15] employed ABM to build a framework of affordance-based human behaviors to simulate the interaction between perception-based dynamic human behaviors and emergency environmental changes (e.g., a sudden fire disaster). Moreover, based on ABM, Wagner and Agrawal [16] proposed a decision support system to simulate crowd evacuation in the event of a fire at a concert venue. It is known from the above research results, that agent-based modeling can be used to define dynamic systems such as agents and related variables, events and states, and behavioral processes to simulate real-world complex behaviors.

Each evacuation process or step is limited by the disaster environment and available resources, so evacuation must be simulated under these constraints to find an approximate best solution that can be performed. To achieve this task for an outpatient chemotherapy area with consideration of the five items mentioned above, this study proposes a constraint-based simulation [17,18,19,20] system with the capability for visual representation. The proposed system comprises three main processes: the definition of spatial and activity constraints, the modeling of agents, and the optimization of additional resource allocations. The spatial boundaries that represent the internal layouts of the chemotherapy area are converted from three-dimensional models developed with building information modeling (BIM). The activity and time restrictions (i.e., the simulation parameters) of regular and evacuation processes in the outpatient chemotherapy area are extracted by employing a work study. Based on spatial boundaries and activity restrictions, this paper employs ABM to construct a visualization system to simulate evacuation processes with different typical process situations. To simulate the stochastic behaviors of nurses when rescuing patients, the Monte Carlo method is adopted to randomly assign rescue tasks to nurses to create possible cooperation in the evacuation process. The results demonstrate that the proposed system can explore the relationships between the activities of chemotherapy patients and nurses based on what meaningful information can be further extracted to effectively allocate resources for evacuation plans. Finally, the proposed system can output visualizations of the data as important information to assist hospital administrators with establishing more realistic and effective emergency response measures in the event of a disaster.

## 3. The Proposed Research Framework

**Figure 1:**
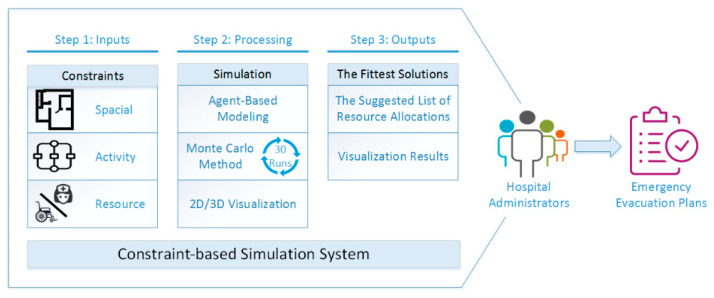
The research framework of the proposed method.

The practical implementation of an emergency evacuation plan is often restricted due to a lack of manpower and equipment resources. Such insufficiencies present a great challenge for hospital administrators in effectively allocating resources to evacuate medical personnel, patients, and their families within the shortest possible time to achieve the target, which is zero injuries and zero deaths. This study proposes a systematic simulation framework to help administrators determine the most appropriate evacuation plan for an outpatient chemotherapy area. As shown in Figure 1, the framework consists of three main processes: constraint construction, simulation, and resource optimization. In the constraints, the spatial boundaries are defined by the layouts converted from an as-built BIM model, while the simulation parameters are decided by the results extracted from the regular and evacuation processes. Based on the constraints, ABM models with allocated resources are built using AnyLogic software (The AnyLogic Company, Saint Petersburg, Russia), for which the computing kernel is the social force models. Then the Monte Carlo method is employed to simulate the stochastic behaviors of nurses in the evacuation process based on an ABM model so as to estimate evacuation time. Finally, based on the simulation results, the system generates a suggested list of resource allocations and visualization results. These data can serve as an important reference for hospital administrators in creating the most appropriate emergency evacuation plans.

## 4. System Constraints

### 4.1. The Spatial Constraints

**Figure 2:**
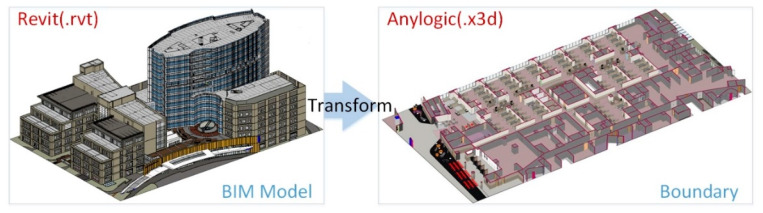
Extracting spatial boundaries from a BIM model for use in the constraint-based simulation.

The emergency evacuation of a hospital is limited by the activity space, which also affects the planning of the overall evacuation plan. This study reuses the BIM model previously established in the design planning stage. In addition, AnyLogic only supports the importation of 3D figures stored in X3D files using a special 3D object element. X3D is a 3D virtual reality modeling language. It defines the encoding format for both the description of 3D models and the description of interactions, commands, and behavioral patterns. From this stage, information such as the activity space, indoor compartments, entrances and exits, and walkway widths is extracted from the BIM model and converted into a virtual reality format X3D file that can be read by the simulation system for conversion into space-limited blocks for later simulation use. Therefore, this study develops a conversion tool for capturing and formatting BIM model data. As shown in Figure 2, the user can directly retrieve the floor plan and compartment data of the BIM model, convert it into the X3D virtual reality file format, and directly import it into a constraint-based simulation system constructed by AnyLogic to convert it to the active space boundary condition of the system. The reuse of the BIM model makes the space condition limitation and simulation visualization more real and reliable.

### 4.2. The Activity Constraints

**Figure 3:**
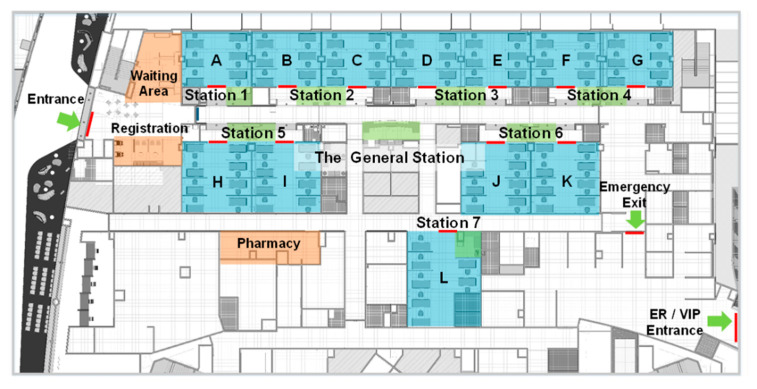
The floor plan of the outpatient chemotherapy area in the cancer medical center.

This study mainly focuses on cancer patients who have been diagnosed by physicians as requiring basic chemotherapy, taking into consideration the uncontrollable factors that occur in complicated situations. To analyze the workflow for the simulation parameters in a general case, a work study (i.e., a motion and time study) on the regular and evacuation processes in an outpatient chemotherapy area is conducted. The area has two exits and four subareas: the registration counter, the waiting area, the pharmacy, and the ward area. As shown in Figure 3, the ward area has twelve treatment rooms (colored blue) and eight nursing stations (colored green). The allocation of nurses and beds is listed in Table 1.

This research simulates the regular working condition of the chemotherapy room as the initial condition before evacuation simulation. When the chemotherapy room is full, the evacuation simulation is carried out. Everyone should put down their work and rescue according to the guidance. This simulation method is more in line with the current situation of the hospital. All simulation parameters and processes are interviewed by actual experts, and these data can be used as important reference basis for related research.

#### 4.2.1. The Work Study of the Regular Processes

**Figure 4:**
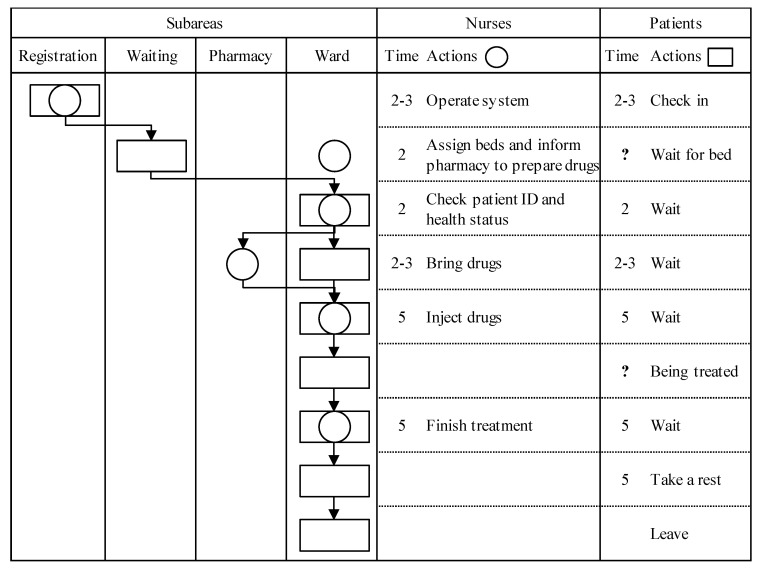
The regular processes for the chemotherapy treatment of a patient.

The work process in the chemotherapy area is analyzed as shown in Figure 4, and the process is briefly explained as follows: At the registration counter, when patients check in, nurses use an information system to order the nurses in the general nursing station to obtain the patient profiles. This process usually takes two to three minutes for each nurse with a patient. After registering, the patients have to wait for beds for chemotherapy in the waiting area; in the meantime, nurses in the general nursing station assign beds to the patients and order the pharmacy to dispense patient prescriptions through the information system. The wait time for a patient is uncertain because it is determined according to the number of available beds in the ward area and the order of the queuing sequence. However, when beds are immediately available, a patient has to wait only about two minutes for a bed. When the general nursing station assigns a bed to a patient, the nurse in charge of the room housing the bed escorts the patient to the bed. After patients enter the ward area, nurses confirm the patients’ identification, double check the correctness of the medicines and doses in the prescriptions, and evaluate the health status of patients, such as blood pressure and body weight, to ensure that the patients can be treated with their prescriptions. When these factors are confirmed as correct, the nurses fasten an identification band onto the patient. The above actions take about two minutes. Then the nurses go to the pharmacy to obtain the medicines for the patients, which typically takes two to three minutes.

When the nurses return with the medicines, the patients begin their chemotherapy. The actions of the nurses, such as equipment disinfection, equipment operation, and drug injection, usually take about five minutes. The treatment time depends on the cancer type and treatment plan and always takes more than thirty minutes. Based on past records, the treatment time ratios are summarized in Table 2. While patients are being treated, nurses can assist other patients with all the actions mentioned above or complete their regular operations and reports at their nursing stations.

When the patients’ treatments are finished, it takes the nurses about five minutes to unplug the treatment equipment and assess the patients’ clinical conditions to decide whether they need additional medical attention or can leave the chemotherapy area. Patients usually need to rest for five minutes to recover before leaving.

#### 4.2.2. The Work Study of the Evacuation Process

To learn the contingency policy adopted for an emergency event in the chemotherapy area, an in-depth interview was conducted with administrators at the hospital. Because the chemotherapeutic drugs used in the area are highly toxic, they must be handled with the utmost care to avoid exposure or damage. Therefore, the nurses first have to terminate the drug injection process for the patients who are under treatment to prevent the people in the area from being affected. It is worth noting that needle withdrawal will prolong the entire evacuation time. Moreover, to avoid drug spillage in emergency situations, administrators typically adopt the drug-carrying escape method in their evacuation plans. Moreover, in an emergency situation, patients are identified as belonging to two groups: those who can be evacuated quickly and can move on their own under guidelines provided by nurses and relief support staff (referred to as medical personnel hereafter), and those who cannot. Some patients are weak and thus cannot move because of their chemotherapy treatments. These patients will require additional resources (e.g., wheelchairs and/or medical personnel) to help them escape from the area. According to the interview, about ten to twenty percent of all patients will need to be evacuated by medical personnel.

The work study examines all possible evacuation actions beginning with the scenario, a stable state in the chemotherapy area as described in Section 4.2.1, when the hospital encounters an emergency event. Two assumptions are made: (1) Medical personnel have been well trained to avoid drug exposure and spillage, and (2) medical personnel understand the evacuation routes well. Two resources are controlled: (1) The number of additional wheelchairs for the use of weak patients, and (2) the number of additional medical personnel who can help weak patients escape from the area.

When the hospital receives an emergency alert, patients at the registration counter and in the waiting area and the patients who have just finished their treatments in the ward area can be guided by medical personnel to escape from the chemotherapy area. In the ward area, it takes medical personnel two-and-a-half to five minutes to terminate drug injections and pack drugs for drug-carrying escape for the patients who are under treatment. Moreover, it takes medical personnel about half a minute to get a wheelchair for a patient.

## 5. The Simulation of Agent-Based Modeling

AnyLogic is adopted in this study as the platform to build the proposed system. Based on the spatial and activity constraints, evacuation conditions for emergency events can be simulated. The computing kernel of AnyLogic software comprises social force models, which enable AnyLogic to simulate complex real-world behavior by defining dynamic systems, such as agents and related variables, events and states, and behavioral processes within a desired timeframe. The simulation model designed for the hospital chemotherapy area can be set as an agent when it has attributes, feedback reactions, and behavioral activities during the simulation process. A multi-agent system consists of multiple independent agents interacting with each other. They can result in different sorts of complex and interesting behavior. It is a method to model real-life situations.

When a hospital encounters an emergency event, hospital administrators will dispatch additional resources to ensure that evacuation plans are implemented well. As mentioned in Section 4.2.2, the controllable factors in this study are the number of additional wheelchairs and the number of additional medical personnel who can help weak patients escape from the chemotherapy area. Such resources, which significantly affect the entire evacuation time, are highly valuable in an emergency because other areas in the hospital may also need these resources. This study develops different resource allocations to perform emergency evacuation simulations. In each simulation run, the proposed system employs the Monte Carlo method to generate random rescue tasks for each nurse according to the spatial and activity constraints.

When an emergency event occurs, the most important action for a medical employee is to stop the injection of medicine for a patient who is under treatment, as mentioned in Section 4.2.2. However, the rescue tasks of medical personnel in the evacuation process are stochastic, for actions will vary in response to different situations. Four assumptions are thus made as follows: (1) Each medical employee stops an injection of medicine and packs medicine for only one patient at a time without assistance, so the patients will be rescued individually; (2) medical personnel must provide wheelchairs for the patients who are too weak to move by themselves; (3) medical personnel can directly escort patients with their packed medicines to escape after the injection is stopped; (4) medical personnel must ensure that the patients in the treatment rooms they are in charge of can be safely evacuated before they begin to help patients in the other rooms.

**Figure 5:**
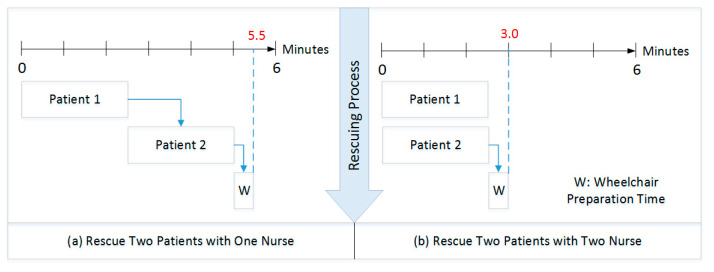
Two stochastic behaviors of nurses will result in two different outcomes.

Obviously, effectively determining manpower allocation can be regarded a non-deterministic polynomial (NP) hard task, for it involves the current numbers of patients and the stochastic behaviors of medical personnel, which may result in different feasible evacuation outcomes [21,22,23]. In Figure 5, if only one nurse assists in the needle extraction process, the second patient needs to wait for the nurse to finish handling the first patient before processing the second patient. If there are two idle nurses, they can handle two patients simultaneously, shortening the evacuation time. However, excessive human resource investment does not necessarily result in equal proportions of benefits, but even leads to wastage of resources.

**Figure 6:**
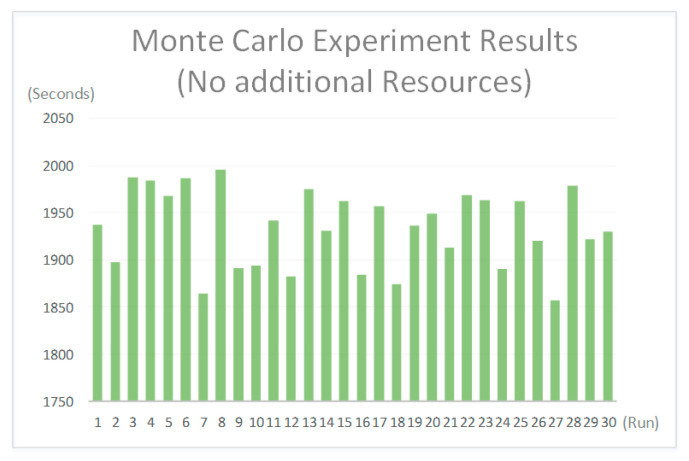
The AnyLogic can collect simulation outputs and view them as a histogram.

To search for the best allocation of manpower and wheelchairs with the shortest evacuation time, the Monte Carlo method [24,25] is employed to achieve this NP-hard task. The Monte Carlo method comprises computational algorithms that rely on repeated random sampling to obtain numerical results, and it can be applied to solve any problem having a probabilistic interpretation. This simulation is constructed with the Monte Carlo experiment functions of AnyLogic. The experiment allows users to run a simulation a number of times, collect outputs, and view them as a histogram, as shown in Figure 6. The proposed system simulates the stochastic behavior of a medical worker (i.e., an agent) by randomly combining working items based on the spatial and activity constraints to create a working list. An agent will thus implement the evacuation process according to the working list. Different resource allocation schemes are assigned in this study to limit the use of the simulation system. After a number of simulations are conducted and the different simulation results are recorded, the best resource allocation scheme can be obtained through result analysis and used as a reference for the emergency evacuation plan.

## 6. Demonstration

This study employs the outpatient chemotherapy area of the cancer medical center as the main research case. First, the spatial relationship of the model in the outpatient chemotherapy area is positioned to facilitate the agent’s judgement on the action. The space in this area consists of the Registration Area, the Waiting Area, Wards A–L, and the Pharmacy, with a total area of approximately 491.1 m^2^, including 12 rooms and 80 beds. In a common situation, eleven nurses are allocated to the outpatient chemotherapy area to maintain regular operations. The remainder of the space is used for the medical staff office, the counseling room, and the medical equipment room, which patients are forbidden to enter. In consideration of the biohazards for patients and medical personnel, the hospital is designed with a single entrance and a single exit, near which all the patients and employees are strictly controlled to prevent congestion. According to the results of the interview, the patients’ diagnoses and treatment ratio are set, and in general, the maximum daily flow number is 280–450, which is the full status of the chemotherapy area.

### 6.1. The Simulation of the Regular Process

The purpose of simulating the regular process is to provide the beginning scenario for the evacuation process simulation. In this study, the simulation of the regular process is run until the system becomes stable with the desired number of occupied beds in order to evaluate the effect on the evacuation plans. In the initial stage, eleven nurses are distributed among the registration counter and the eight nursing stations, where they are preparing to serve patients in the chemotherapy area. In addition, seven wheelchairs are deposited in the equipment room. Although the proposed system is the type of model used in the fuzzy queuing theory [26], in which the customers’ arrival rate has a Poisson distribution but the service rate is fuzzy, the patients will arrive according to appointments that were scheduled in advance to avoid long waiting times. According to past records, patient arrival numbers are highest at opening time and then decrease thereafter. At opening time, the eleven nurses, who are initially idle, will engage in operations at the registration counter and in the ward area to quickly stabilize the system. When a patient arrives at the registration counter, the proposed system performs a simulation according to the process listed in Figure 4. In addition, Table 3 also lists the movement speeds of the patients, nurses, and wheelchairs, and the ratio of patients who come with wheelchairs. The walking speed of patients and the traveling speed of the wheelchairs are set as obeying uniform distributions (0.8, 1) and (0.5, 0.8), respectively, to represent the physical statuses of the patients.

**Figure 7:**
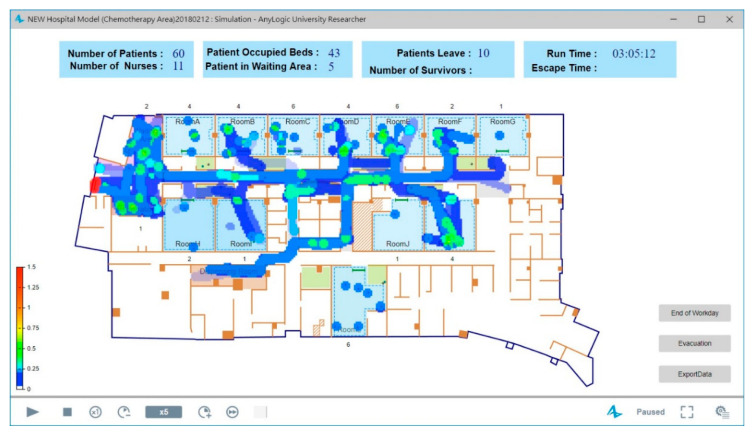
The simulation results for the regular process with pedestrian density.

**Figure 8:**
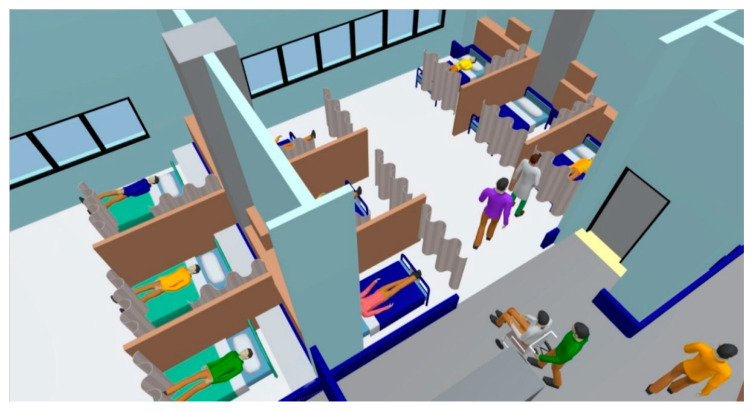
The proposed system constructs three-dimensional perspectives to represent the activities of patients and nurses utilizing BIM models.

The simulation results are shown in Figure 7, which provides the number of patients, the number of nurses, the number of occupied beds, the number of patients in the waiting area, and the number of patients leaving. In addition to being the basis on which the spatial constraints in a hospital are represented, the use of the floor plan converted from a BIM model has the benefit of directly showing the pedestrian density at a given hour. The routes walked by more pedestrians will be colored close to red. For example, in the simulation time, the routes that have no pedestrians, few pedestrians, and the most pedestrians will be colored white, blue, and red to show no density, low density, and high density, respectively. The color red denotes the critical density, for which the default value is set as 1.5 pedestrians/m^2^. Hospital administrators thus can quickly understand the utilization rate of the space for further planning, since an overcrowded chemotherapy space will affect service quality [27]. Furthermore, the proposed system can provide more realistic simulation processes for administrators by importing the three-dimensional aspects converted from BIM models. As in the diagram shown in Figure 8, all the activities of patients and nurses, such as waiting, walking, and treatment, can be monitored in real time.

### 6.2. The Simulation of the Evacuation Process

The objective of simulating an evacuation process is to understand the possible evacuation results when a disaster occurs in a hospital. This simulation begins with the stable state in the regular process, and the behavior modes of all agents in AnyLogic become the emergency mode, including the escape routes of patients and medical personnel, the rescue mechanisms of medical personnel, and the assistance of resources.

In this paper, we assume that an emergency event occurs and blocks the entrance of the chemotherapy area, so all patients and medical personnel must evacuate from the only exit according to the escape principles. The patients who are undergoing treatment must wait for medical personnel to stop their injections and pack their drugs for transportation. Eleven nurses and seven wheelchairs are allocated as the initial resources, and the remainder of the simulation parameters are listed in Table 4. Although a weak patient can escape with the help of a nurse without using a wheelchair and the escape speed is faster (1.5 m/s), the manpower of a nurse who can rescue other patients undergoing treatment is lost until the patient has escaped.

**Figure 9:**
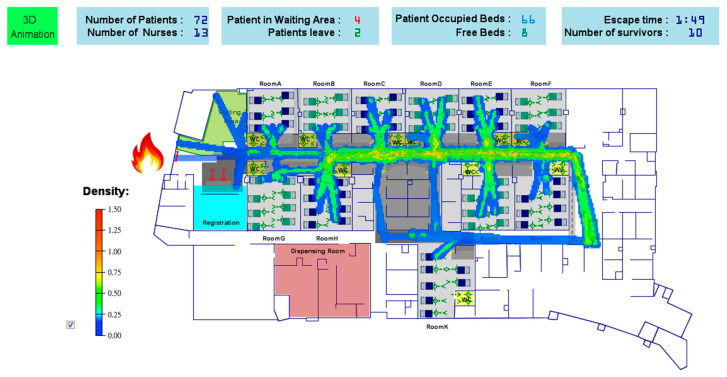
The evacuation process simulation results with pedestrian density.

When the hospital sounds the alarm, the proposed system will automatically record the total evacuation time until all patients and medical personnel have successfully escaped from the chemotherapy area. This research considers different patient rescue procedures according to their response and condition. These results will affect the evacuation simulation time. A simulation result of the situation of one hundred percent of the beds occupied with the eleven nurses and seven wheelchairs is shown in Figure 9. By employing the Monte Carlo method to simulate the stochastic rescue tasks of the nurses, the maximum evacuation time derived from thirty simulation runs is 33 min and 15 s, which exceeds the hospital requirement of 20 min. Accordingly, hospital administrators must dispatch additional resources, nurses, and wheelchairs to the area to reduce the evacuation time. Further experimental results with different percentages of occupied beds and resource allocations are discussed in the next section.

### 6.3. Monte Carlo Method for Finding the Fittest Solution

**Figure 10:**
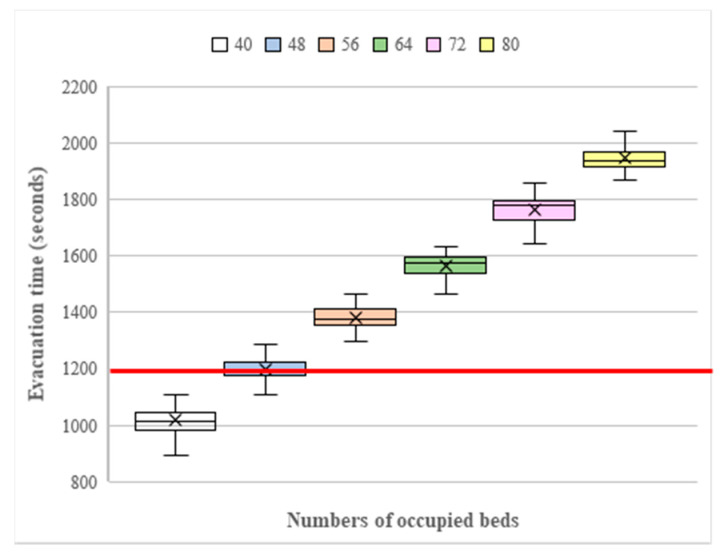
The evacuation times for different numbers of beds occupied without additional resources.

The goal of the simulation is to create a list of evacuation times with different resource allocations as a reference for hospital administrators to create the best evacuation plan for the outpatient chemotherapy area. In the following subsections, the experimental settings and results are discussed. Since a bed being in use means medical personnel will need two-and-a-half to five minutes to stop the drug injection and pack drugs for the patient, the number of occupied beds will significantly affect the total evacuation time. Therefore, in this work, a sensitivity analysis is conducted to examine the effects of the number of beds occupied by patients and the number of additional resources issued by administrators on the evacuation time. Therefore, the experiment involves three factors: the number of occupied beds, the number of additional nurses, and the number of additional wheelchairs. Given that the initial resource allocation is eleven nurses and seven wheelchairs and that the maximum acceptable evacuation time is twenty minutes (i.e., 1200 s), and taking into consideration the stochastic results from the Monte Carlo method, each simulation with a set of parameter combinations is performed thirty times. The final evacuation time is represented by the maximum evacuation time to ensure that the most loosely stochastic behavior of the nurses can still meet the maximum acceptable evacuation time when a hospital encounters an emergency event. To determine the range of beds occupied by patients to evaluate the evacuation time in the experiments, a pre-test is conducted without any additional resources. Each thirty simulation runs for each number of occupied beds is drawn in box-and-whisker plots to show the distributions in Figure 10. It can be observed that when forty beds are occupied, the total evacuation time for the thirty simulation runs is less than 1200 s. The results indicate that with the initial resources, eleven nurses and seven wheelchairs, the evacuation process can be completed within 1200 s when the number of occupied beds is less than or equal to fifty percent of all beds. Accordingly, the experiments will begin using a total of forty-eight beds.

The experimental results representing the maximum evacuation time in each of the thirty simulation runs are summarized in Table 5. Although the maximum value (evacuation time) is not a stable value, as would be the case for a mean or a median, it refers to the most loosely stochastic behavior of the nurses and thus ensures the highest standard when developing an evacuation plan. The additional resources suggested for each number of occupied beds are also highlighted in gray in Table 5. For each number of occupied beds, two suggestions with different numbers of nurses and wheelchairs are provided. The suggestion is determined based on the three conditions with the following priorities: (1) The evacuation time must be less than 1200 s; (2) the minimum requested resources should be dispatched; (3) the nurses and wheelchairs must be interchangeable. The additional resources for different numbers of occupied beds are suggested as follows: Forty-eight occupied beds require two nurses with zero wheelchairs or one nurse with one wheelchair; fifty-six occupied beds require four nurses with zero wheelchairs or three nurses with one wheelchair; sixty-four occupied beds require six nurses with zero wheelchairs or five nurses with two wheelchairs; seventy-two occupied beds require eight nurses with zero wheelchairs or seven nurses with three wheelchairs; eighty occupied beds require ten nurses and zero wheelchairs or nine nurses and one wheelchair.

In addition, the averaged margins of nurses and wheelchairs are provided to show the marginal effect on the two factors. The averaged margins of the nurses clearly show that the effectiveness of the plan decreases following the law of diminishing marginal utility [28]. According to the experimental results, the additional manpower of a single nurse mostly reduces the entire evacuation time. Moreover, since nurse manpower effectiveness decreases following the law of diminishing marginal utility, dispatching more nurses to the area makes it progressively less likely that the total evacuation time will be further reduced. In contrast, the averaged wheelchair margins do not. This is true for two reasons. One is that a nurse can substitute for a wheelchair to help a weak patient escape from the outpatient chemotherapy area or when the patient is near the exit. The other is that patients who are unable to escape by themselves normally bring their own wheelchairs to the outpatient visit, so they will use their own wheelchairs to escape when the emergency event occurs, and no additional wheelchair resources are needed. If a patient without a wheelchair experiences discomfort during the chemotherapy process, additional wheelchair resources are needed to assist the patient to move. Increasing the number of wheelchairs will not obviously increase the evacuation efficiency.

## 7. Conclusions

Ensuring that there are no deaths when a hospital is faced with a sudden disaster is always a high priority for hospitals. Although developing emergency evacuation plans and sufficient drilling can overcome this issue, plans should be rechecked or revised whenever internal layouts change. This is especially true for a chemotherapy area, where the highly toxic drugs used must be handled with the utmost care to avoid placing patients at risk. When a disaster occurs, patients who are undergoing treatment will need medical personnel to stop their drug injections and pack their drugs for drug-carrying escape. In addition, patients who are unable to move after their treatments will need wheelchairs or medical personnel to help them escape from the area. The above conditions will greatly prolong the entire evacuation time.

To help administrators predict possible evacuation results based on different resource allocations before they determine the final evacuation plan for an outpatient chemotherapy area, this paper uses agent-based modeling and BIM technology to construct a constraint-based simulation to simulate the general situation and emergency evacuation of a normal outpatient chemotherapy area. Agent-based modeling can be used to define dynamic systems to simulate real-world complex behaviors. Moreover, the BIM model built during the design phase is reused to provide information on the geometric appearance, spatial location and building materials required for the simulation. The group behaviors and conditions of complex emergency evacuations in hospitals can thus be visualized in an immersive environment. Finally, to simulate the stochastic behavior of nurses in an evacuation process, the Monte Carlo method is employed to randomly order the rescue actions of the nurses (agents), since the cooperation of nurses will shorten the total evacuation time.

The allocation of too many resources at normal times may result in waste of labor and increased labor costs. In contrast, if too few resources are allocated, the hospital administration might not be able to cope with an emergency. Therefore, this study focuses on the simulation of the resource allocation relationship in the outpatient chemotherapy area during an emergency evacuation. In this study, the initial resources allocated in the outpatient chemotherapy area included eleven nurses and seven wheelchairs. When the hospital encounters a disaster, administrators have to dispatch additional nurses and wheelchairs to the area to ensure that everyone can be evacuated within twenty minutes. The factors that affect the evacuation time are the number of beds occupied, the number of nurses, and the number of wheelchairs. For each combination of the three factors, simulation was performed thirty times, and the maximum evacuation time was taken to illustrate the result. The experimental results demonstrated that the proposed system can successfully create a suggested list of additional resources for five different numbers of occupied beds with the five considerations mentioned in Section 1. Moreover, the simulation results can be visualized with 2D and 3D methods, which provide data for planning and decision-making personnel as an important reference. Hospital administrators can determine their final evacuation plans according to the suggested list of resource allocation and visualization results.

Disasters cannot be completely prevented, but it is hoped that the goal of zero casualties can be achieved through different means. This study only simulates the relevant personnel in the hospital’s chemotherapy area, but everyone should put down their work and rescue according to the guidance when the disaster occurs. In the future, if the system wants to simulate the evacuation more accurately, the status and the related parameters of other personnel should be included in the simulation to make the simulation in line with the current situation. These results are important reference for administrators to make evacuation plans for achieving the goal of zero casualties.

## Figures and Tables

**Table 1:** The allocation of nurses and beds in the ward area.

Nursing Stations	Nurses	Rooms	Bed Capacity
1, 2, 3, 4, and 7	1	A, B, C, D, E, F, G	6
5 and 6	2	H, I, and K	8
The general nursing station	1	J and L	7
The registration counter	1		
Total	11	Total	80

**Table 2:** The ratios of different treatment times for a patient.

Treatment Time (Minutes)	Ratio (%)
30–60	25
60–120	30
180–240	35
>480	10

**Table 3:** Simulation parameters for the regular process.

Parameter	Value
Patient walking speed	Uniform (0.8, 1) m/s
Nurse walking speed	0.8 m/s
Wheelchair speed	Uniform (0.5, 0.8) m/s
Ratio of patients using wheelchairs	10–20%

**Table 4:** Simulation parameters for evacuation process.

Parameter	Value
Patient self-escape speed	1.28 m/s
Nurse working/self-escape speed	1.5 m/s
Escape speed with assistance from nurses with wheelchairs	Uniform (1, 1.28) m/s
Wheelchair preparation time	0.5 min
Time needed to terminate injections during an emergency	2.5–5 min
Percentage of patients who need wheelchairs	10–20%

**Table 5:** The maximum evacuation times for the thirty simulation runs.

Occupied Beds	Additional Nurses	Additional Wheelchairs					Averaged Nurse Margin
		0	1	2	3	4	
48	0	1264.0	1272.0	1273.0	1257.0	1275.0	
	1	1210.0	1197.0	1227.0	1200.0	1178.0	65.8
	2	1128.0	1132.0	1112.0	1131.0	1129.0	76.0
	3	1042.0	1026.0	1054.0	1035.0	1053.0	84.4
	4	991.0	983.0	966.0	1014.0	979.0	55.4
	Averaged wheelchair margins		5.0	−4.4	−1.0	4.6	
56	0	1481.0	1472.0	1470.0	1447.0	1453.0	
	1	1373.0	1345.0	1344.0	1365.0	1349.0	109.4
	2	1252.0	1305.0	1246.0	1261.0	1298.0	82.8
	3	1212.0	1168.0	1174.0	1185.0	1182.0	88.2
	4	1107.0	1115.0	1130.0	1138.0	1175.0	51.2
	5	1076.0	1066.0	1105.0	1070.0	1079.0	53.8
	Averaged wheelchair margins		5.0	0.3	0.5	−11.7	
64	0	1639.0	1656.0	1667.0	1675.0	1639.0	
	1	1525.0	1525.0	1514.0	1552.0	1516.0	128.8
	2	1450.0	1411.0	1438.0	1429.0	1444.0	92.0
	3	1334.0	1359.0	1335.0	1358.0	1335.0	90.2
	4	1283.0	1274.0	1234.0	1260.0	1259.0	82.2
	5	1218.0	1209.0	1199.0	1185.0	1187.0	62.4
	6	1152.0	1118.0	1137.0	1164.0	1149.0	55.6
	7	1076.0	1079.0	1097.0	1079.0	1089.0	60.0
	Averaged wheelchair margins		5.8	1.3	−10.1	10.5	
72	0	1842.0	1860.0	1849.0	1858.0	1801.0	
	1	1708.0	1735.0	1717.0	1713.0	1688.0	129.8
	2	1623.0	1594.0	1622.0	1609.0	1609.0	100.8
	3	1481.0	1500.0	1471.0	1502.0	1540.0	112.6
	4	1409.0	1429.0	1396.0	1384.0	1439.0	87.4
	5	1329.0	1319.0	1318.0	1345.0	1338.0	81.6
	6	1275.0	1250.0	1283.0	1255.0	1272.0	62.8
	7	1202.0	1206.0	1200.0	1183.0	1218.0	65.2
	8	1171.0	1175.0	1140.0	1136.0	1126.0	52.2
	9	1088.0	1073.0	1115.0	1111.0	1099.0	52.4
	Averaged wheelchair margins		−1.3	3.0	1.5	−3.4	
80	0	1995.0	2046.0	2025.0	1996.0	2007.0	
	1	1909.0	1895.0	1913.0	1920.0	1854.0	115.6
	2	1750.0	1782.0	1711.0	1715.0	1745.0	157.6
	3	1636.0	1636.0	1620.0	1648.0	1655.0	101.6
	4	1536.0	1553.0	1531.0	1547.0	1524.0	100.8
	5	1485.0	1462.0	1452.0	1475.0	1425.0	78.4
	6	1379.0	1381.0	1370.0	1353.0	1371.0	89.0
	7	1291.0	1301.0	1331.0	1312.0	1292.0	65.4
	8	1262.0	1248.0	1247.0	1252.0	1241.0	55.4
	9	1207.0	1198.0	1186.0	1197.0	1204.0	51.6
	10	1137.0	1144.0	1149.0	1149.0	1161.0	50.4
	11	1102.0	1087.0	1140.0	1115.0	1107.0	37.8
	Averaged wheelchair margins		−3.7	4.8	−0.3	7.8	

Gray shows the suggestions of resource allocation.
